# The Separation of the Mn_12_ Single-Molecule Magnets onto Spherical Silica Nanoparticles

**DOI:** 10.3390/nano9050764

**Published:** 2019-05-18

**Authors:** Lukasz Laskowski, Iwan Kityk, Piotr Konieczny, Oleksandr Pastukh, Mateusz Schabikowski, Magdalena Laskowska

**Affiliations:** 1 Institute of Nuclear Physics Polish Academy of Sciences, PL-31342 Krakow, Poland; lukasz.laskowski@ifj.edu.pl (L.L.); piotr.konieczny@ifj.edu.pl (P.K.); oleksandr.pastukh@ifj.edu.pl (O.P.); mateusz.schabikowski@ifj.edu.pl (M.S.); 2 Institute of Optoelectronics and Measuring Systems, Faculty of Electrical, Engineering, Czestochowa University of Technology, Armii Krajowej 17, PL-42200 Czestochowa, Poland; iwank74@gmail.com

**Keywords:** single-molecule magnet, Mn_12_, interactions, silica

## Abstract

The Mn_12_ single-molecule magnets (SMMs) could be attached to the surface of spherical silica for the first time with a high probability. This allowed separation of the individual molecular magnets and direct microscopic observation of the SMMs. We described in detail how to fabricate such a composite material. The synthesis procedure proposed here is simple and efficient. We confirmed the efficiency of the method by transmission electron microscopy (TEM): single-molecule magnets were visible at the surface of a silica substrate. Based on TEM observation, we described how the molecules anchor to the surface of silica (the geometry of the magnetic molecule in regard to the surface of the substrate). The SQUID magnetometry showed that single-molecule magnet behaviour is kept intact after grafting. The attachment of the single-molecule magnets to the surface of silica allows to investigate their properties as separate molecules. This is particularly important in the analysis of magnetic properties such as magnetic states of the separated SMMs, their mutual interactions, and the influence of a silica support.

The first synthesis of single-molecule magnets (SMMs, Mn${}_{12}$ac${}_{16}$) was described by Lis et al. in 1980 [1]. Nevertheless, their unusual behaviour was observed for the first time in the 90s [2]. Since then, SMMs are in the main field of interest of many scientific groups. Their individual molecules behave as a ferromagnet below blocking temperature. Although a lot of other types of SMMs have been synthesized since then, Mn${}_{12}$ac${}_{16}$ is still interesting for scientists because of its high intrinsic spin (S = 10) and a slow relaxation of magnetization [3]. Moreover, the synthesis of these magnets is uncomplicated and low-cost. However, one of the most important problems related to this material is the investigation of its individual molecules because Mn${}_{12}$ac${}_{16}$ is practically insoluble in commonly available solvents [4]. This makes the separation of individual particles of this compound difficult to perform. There were a few attempts to solve this task. However, a successful separation of the magnetic molecules has not been unambiguously proven [5,6,7]. Even by using sophisticated equipment for Mn${}_{12}$ac${}_{16}$ dilution, molecules still seem to be agglomerated in clusters [8]. A detailed discussion of this problem can be found in the literature [9]. In our last work, we anchored Mn${}_{12}$-based SMMs inside SBA-15 silica pores with promising results. Nevertheless, individual molecules could not be directly observed [9].

Here, we propose a novel, simple, and efficient procedure that allows for the separation of Mn${}_{12}$ac${}_{16}$-based single-molecule magnets. The individual molecules can be easily observed with the use of transmission electron microscopy (TEM). The key idea is to anchor single-molecule magnets to the surface of spherical silica nanoparticles using propyl carbonic acid groups, as shown in Figure 1. The spherical shape of the silica support allows for a very precise microscopic observation making the individual single-molecule magnets clearly visible. Moreover, due to the relatively large specific surface area (13 m${}^{2}$/g), the number of immobilized SMMs is sufficient to precisely investigate their magnetic properties as separated molecules. Furthermore, the distance between magnetic molecules can be finely tuned with the use of the method of distribution control [10].

In the first step, we prepared spherical silica nanoparticles according to the Stöber protocol [11]. Obtained silica spheres (further denoted as **Sil-S**), with a diameter of 300 nm, were grafted with butyronitrile units. To this end, we prepared the solution of 4-(triethoxysilyl)butyronitrile (BNTES) in dichloromehane (2% of volume). The 0.5 g of Sil-S was subsequently added to this solution and mixed under reflux overnight with an applied argon protective atmosphere. Next, the product was recovered by centrifugation and washed with the use of pure dichloromethane. The procedure was repeated four times and the resulting powder was dried under vacuum. In order to avoid unwanted side reactions between carbonic acid groups and surface hydroxyl units during the hydrolysis, the pre-functionalized silica powder was silylated by the solution of chlorotrimethyl silane (ClTMS) in dichloromethane (4% of volume), similarly to the procedure above.

In the next step, cyano units at the end of butyronitrile groups were hydrolyzed into carbonic acid by a solution of HCl (6 M) in water and acetone (1:1 of volume). The reaction was performed under reflux overnight. The resulting powder was centrifuged and washed by acetone (four times, until neutral pH was reached).

Finally, silica-containing carbonic acid anchoring units at the surface were functionalized with Mn${}_{12}$-stearate—a soluble derivative of Mn${}_{12}$ac${}_{16}$. The synthesis of Mn${}_{12}$-stearate was performed according to the protocol described previously [4,12]. For the functionalization of 0.3 g of silica, we applied 0.1 g of Mn${}_{12}$-stearate. Reagents were mixed in dichloromethane overnight at room temperature under an argon protective atmosphere. The resulting powder (**Sil-S-Mn${}_{12}$**) was centrifuged, washed by dichloromethane five times, dried under vacuum for 10 h, and stored in the refrigerator in an argon atmosphere.

The synthesis yields 0.25 g of the final material from 0.5 g of the initial spherical silica. The procedure was illustrated in Figure 2.

In order to confirm the success of the synthesis, we analysed the obtained material (Sil-S-Mn${}_{12}$) under TEM microscope and compared it to the pure spherical silica (Sil-S). The resulting images can be seen in Figure 3.

In the case of both materials, Sil-S-Mn${}_{12}$ and Sil-S, the particles look like clear homogeneous spheres at low magnification. Nevertheless, individual molecules of SMMs can be unambiguously observed in the form of islands on the surface of the silica at higher magnification. We did not observe the agglomeration of Mn${}_{12}$-stearate (also X-Ray reflectivity did not shown any crystalline peaks, typical for the bulk Mn${}_{12}$—see: Supplementary Materials). The molecules can be seen particularly clear at the horizon of the spheres (the border of a planar projection of silica spheres in the images). The single-molecule magnets do not closely adhere to the silica surface but are separated from it. This detachment is probably caused by propyl-carbonic acid anchoring units which separate SMMs from the support. The separation is particularly important for single-molecule magnets. This is because when they are closely adjacent to the substrate, they can, in most cases, lose their magnetic properties [13,14].

In order to additionally confirm if SMMs are anchored at the silica surface via propyl-carbonic acid groups, as we assume, we tried to functionalize the pure spherical silica (with no anchoring units) by the magnetic molecules (application of only step III of the synthesis procedure to the spherical silica). The resulting material did not contain any visible (under TEM) molecules at the surface (see: Supplementary Materials). On this basis, we could conclude that the Mn${}_{12}$-stearate molecules were anchored at the silica surface via propyl-carbonic acid groups with a high probability

The size and geometry of Mn${}_{12}$-stearate molecules can be easily estimated from the TEM picture (Figure 3) on the basis of a linear histogram. They are visible as ellipsoids, with the longer axis between 2.5 and 3 nm whereas the shorter axis was estimated to be approximately 1 nm. These dimensions are in accordance with the theoretical size of an individual molecule of Mn${}_{12}$-stearate: a shape that can be inscribed into a flattened ellipsoid with dimensions of 1.5, 2.5, and 3 nm depending on the observation angle (Figure 4a). The geometry of Mn${}_{12}$-stearate differs from the Mn${}_{12}$ac${}_{16}$, due to long stearic acid chains.

It is worth emphasizing that SMMs are attached to the surface via propyl-carbonic acid link in an umbrella-like arrangement. That is when the largest surface plane of the SMMs molecule is perpendicular to the silica surface, as it was depicted in Figure 4b. In our opinion, such a finding is very important for the analysis of interactions between single-molecule magnets. This observation was possible for the first time due to the use of the spherical silica support.

In order to evaluate whether or not the properties of Mn${}_{12}$-stearate single-molecule magnets remain unchanged after grafting to the surface of silica, we measured the dependence of magnetization on magnetic field for the investigated materials by SQUID magnetometry. The obtained results can be seen in Figure 5.

The material showed visible hysteresis with a coercive field of about 1000 Oe. Moreover, the hysteresis is of a butterfly-like shape, which is typical for single-molecule magnets. Such properties originate from quantum tunneling of magnetization [3]. On this basis we state that the Mn${}_{12}$-st molecules could maintain their SMM behavior even in the form of separated molecules anchored to the surface of silica. The detailed analysis of the magnetic interactions between Mn${}_{12}$-st molecules is the subject of another of our works.

In summary, we have presented a very robust procedure allowing for the separation of Mn${}_{12}$-based single-molecule magnets. The procedure was based on anchoring the individual SMMs onto the silica support. By using spherical silica, it was possible to directly observe separate magnetic molecules in their umbrella-like arrangement with the use of TEM. The SQUID magnetometry showed that the properties of single-molecule magnets can be maintained after separation at the surface of silica. The procedure could have an applicative potential in the investigation of the magnetic properties of single-molecule magnets since they can be finally measured for separate molecules, not many molecules simultaneously. Moreover, by using spacer units [10], the distance between SMMs could be finely tuned and their mutual interactions can be examined.

## Figures and Tables

**Figure 1:**
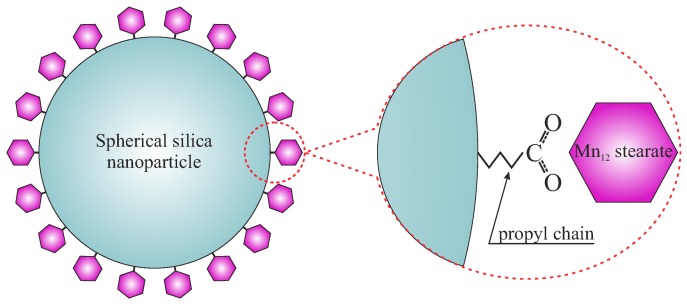
A schematic representation of the proposed material.

**Figure 2:**
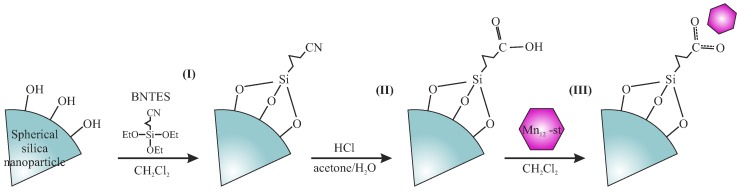
A schematic presentation of the synthesis procedure allowing for the separation of the Mn${}_{12}$ single-molecule onto spherical silica nanoparticles. Assumed steps: grafting (I), hydrolysis (II), and functionalization (III).

**Figure 3:**
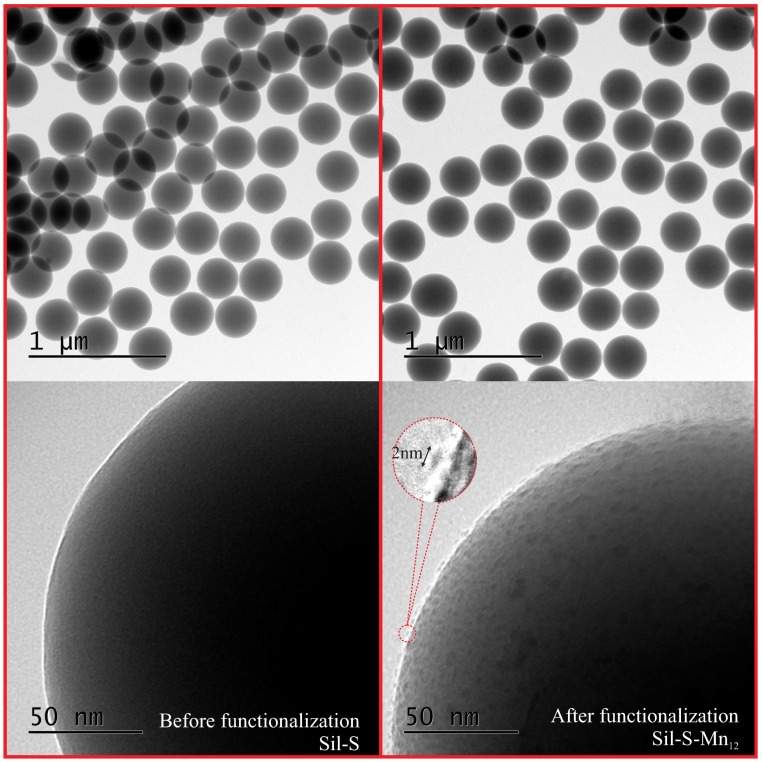
The transmission electron microscopy images of the composite material: individual Mn${}_{12}$-stearate molecules attached to the spherical silica surface (Sil-S-Mn${}_{12}$—right side) in comparison with the pure spherical silica nanoparticles (Sil-S—left).

**Figure 4:**
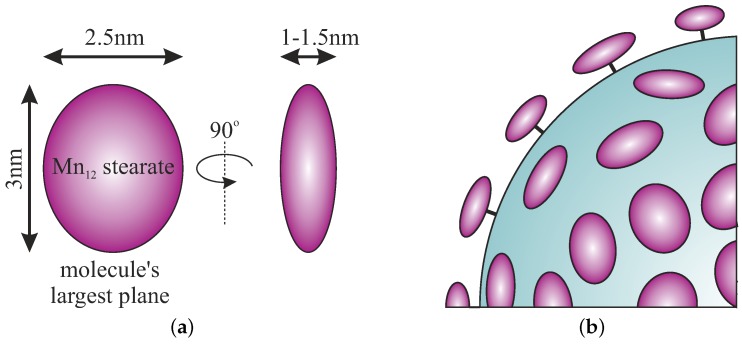
Schematic representation of the geometry of Mn${}_{12}$-stearate single-molecule magnets attached to the surface of spherical silica: simplified geometry of Mn${}_{12}$-stearate molecule (**a**) and the geometry of molecules anchored to the silica support (**b**).

**Figure 5:**
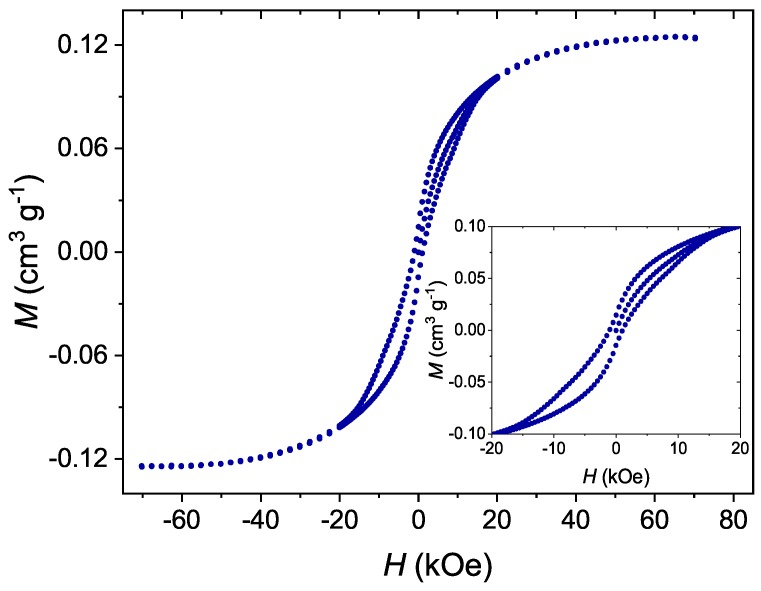
The dependence of magnetization on magnetic field for Mn${}_{12}$-stearate single-molecule magnets attached to the surface of spherical silica measured at a temperature of 2 K.

## Supplementary Materials

The following are available at https://www.mdpi.com/2079-4991/9/5/764/s1, Figure S1: The experiment verifying the role of anchoring units: the transmission electron microscopy images of the materials at different stages of the synthesis and with the application of different steps of the procedure. Pure spherical silica (a), pure spherical silica functionalized by Mn${}_{12}$-st (b), silica with carbonic acid units (c), silica with carbonate acid units functionalized by Mn${}_{12}$-st (d), Figure S2: X-ray diffraction results for the spherical silica containing Mn${}_{12}$-st molecules at the surface.

## Abbreviations

The following abbreviations were used in this manuscript:

SMM	single-molecule magnet
Mn${}_{12}$ac${}_{16}$	$\left[{\mathrm{Mn}}_{12}O_{12}{\left({\mathrm{CH}}_{3}{\left({\mathrm{CH}}_{2}\right)}_{16}{\mathrm{CO}}_{2}\right)}_{11}{\left({\mathrm{CH}}_{3}{\mathrm{CO}}_{2}\right)}_{5}{\left(H_{2}O\right)}_{4}\right]\cdot 2{\mathrm{CH}}_{3}\mathrm{COOH}$
Mn${}_{12}$-st	derivative of Mn${}_{12}$ containing strearic acid ligands
BNTES	4-(triethoxysilyl)butyronitrile

## References

[1] Lis T. (1980). Preparation, structure, and magnetic properties of a dodecanuclear mixed-valence manganese carboxylate. Acta Crystallogr. Sect. B Struct. Crystallogr. Cryst. Chem..

[2] Sessoli R., Gatteschi D., Caneschi A., Novak M. (1993). Magnetic bistability in a metal-ion cluster. Nature.

[3] Christou G., Gatteschi D., Hendrickson D.N., Sessoli R. (2000). Single-molecule magnets. MRS Bull..

[4] Park C.D., Jeong D.Y. (2001). Soluble Single-Molecule Magnet: Mn_12_-stearate. Bull. Korean Chem. Soc..

[5] Willemin S., Arrachart G., Lecren L., Larionova J., Coradin T., Clérac R., Mallah T., Guérin C., Sanchez C. (2003). Immobilisation of single molecule magnets in mesoporous silica hosts. New J. Chem..

[6] Clemente-León M., Coronado E., Forment-Aliaga A., Amorós P., Ramírez-Castellanos J., González-Calbet J.M. (2003). Incorporation of Mn 12 single molecule magnets into mesoporous silica. J. Mater. Chem..

[7] Clemente-León M., Coronado E., Forment-Aliaga A., Martínez-Agudo J., Amorós P. (2003). Mn_12_ single-molecule magnets incorporated into mesoporous MCM-41 silica. Polyhedron.

[8] del Carmen Giménez-López M., Moro F., La Torre A., Gómez-García C.J., Brown P.D., Van Slageren J., Khlobystov A.N. (2011). Encapsulation of single-molecule magnets in carbon nanotubes. Nat. Commun..

[9] Laskowska M., Bałanda M., Fitta M., Dulski M., Zubko M., Pawlik P., Laskowski Ł. (2019). Magnetic behaviour of Mn_12_-stearate single-molecule magnets immobilized inside SBA-15 mesoporous silica matrix. J. Magn. Magn. Mater..

[10] Laskowska M., Oyama M., Kityk I., Marszalek M., Dulski M., Laskowski L. (2019). Surface functionalization by silver-containing molecules with controlled distribution of functionalities. Appl. Surf. Sci..

[11] Stöber W., Fink A., Bohn E. (1968). Controlled growth of monodisperse silica spheres in the micron size range. J. Colloid Interface Sci..

[12] Verma S., Verma A., Srivastava A.K., Gupta A., Singh S.P., Singh P. (2016). Structural and magnetic properties of Mn_12_-Stearate nanomagnets. Mater. Chem. Phys..

[13] Barra A.L., Bianchi F., Caneschi A., Cornia A., Gatteschi D., Gorini L., Gregoli L., Maffini M., Parenti F., Sessoli R. (2007). New Single-Molecule Magnets by Site-Specific Substitution: Incorporation of “Alligator Clips” into Fe4 Complexes. Eur. J. Inorg. Chem..

[14] Mannini M., Pineider F., Sainctavit P., Danieli C., Otero E., Sciancalepore C., Talarico A.M., Arrio M.A., Cornia A., Gatteschi D. (2009). Magnetic memory of a single-molecule quantum magnet wired to a gold surface. Nat. Mater..

