# Supplementary material for: The Separation of the Mn12 Single-Molecule Magnets onto Spherical Silica Nanoparticles
Source: Nanomaterials (Basel). 2019 May 18;9(5):764. doi: 10.3390/nano9050764 (PMC6567163; doi:10.3390/nano9050764)
Supplement: Supplementary file 1 [file nanomaterials-09-00764-s001.pdf]

The Separation of the Mn_{12} Single-Molecule Magnets onto Spherical Silica Nanoparticles

Supplementary Materials

Lukasz Laskowski, Iwan Kityk, Piotr Konieczny, Oleksandr Pastukh, Mateusz Schabikowski and Magdalena Laskowska

Additional experiments

In order to additionally confirm, if SMMs are anchored at the silica surface via propyl-carbonic acid groups, as we assume, we carried out an additional experiment: functionalization the pure spherical silica (with no anchoring units) by magnetic molecules. Obtained results can be seen in the Figure S1 juxtaposed with the results of the functionalization of the silica-containing anchoring units.

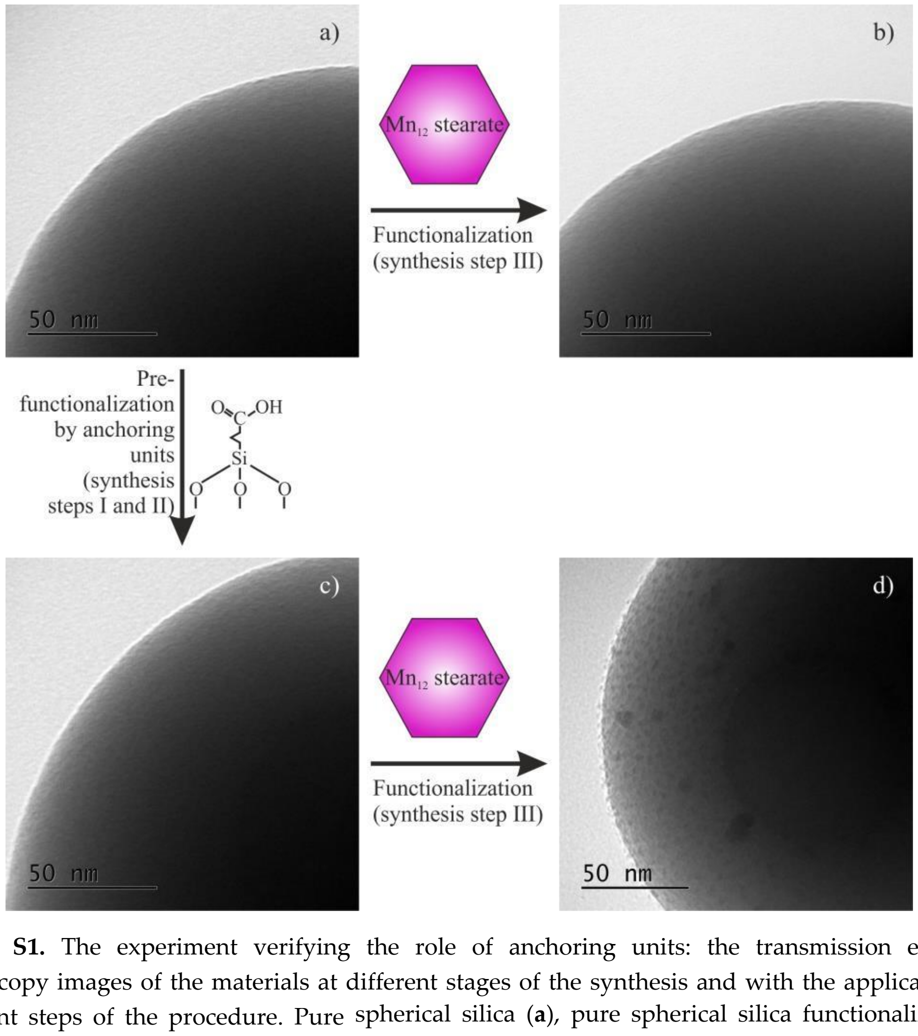

Figure S1. The experiment verifying the role of anchoring units: the transmission electron microscopy images of the materials at different stages of the synthesis and with the application of different steps of the procedure. Pure spherical silica (**a**), pure spherical silica functionalized by Mn_{12}-st (**b**), silica with carbonic acid units (**c**), silica with carbonate acid units functionalized by Mn_{12}-st (**d**).

As can be seen in Figure S1b, resulting material did not contain any visible (under TEM microscope) molecules at the surface. On the contrary, the material possessing carbonic acid anchoring units was successfully functionalized: the surface seems to be covered by Mn_{12}-st

molecules. On this basis, we can conclude, that the carbonic acid groups bonded SMMs and the Mn_{12}-st molecules are anchored at the silica surface via propyl-carbonic acid groups with a high probability.

We carried out the X-Ray reflectivity measurements in order to check if the resulting specimen is free from the bulk agglomeration (crystalline) of the Mn_{12}-st. Results can be seen in Figure S2.

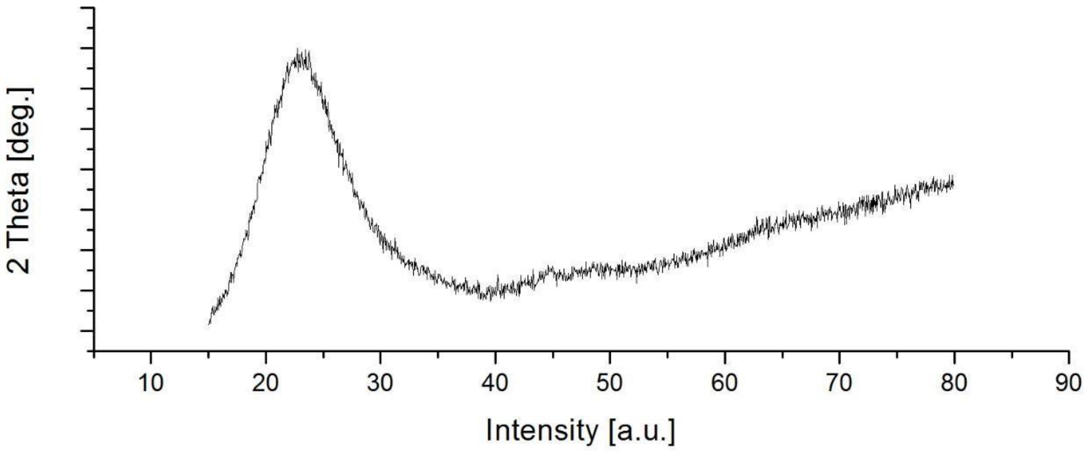

Figure S2. X-ray diffraction results for the spherical silica containing Mn_{12}-st molecules at the surface.

The X-ray showed the plot typical for amorphous silica with no crystalline reflections. However, such a result was in full accordance with our assumption: we did not expect any crystalline phases, only separated Mn_{12} molecules.

Additionally, we carried out EDX elemental analysis and Raman spectroscopy. The amount of the Mn_{12}-st species was too low to detect it by EDX. Raman spectroscopy showed the features originating only from stearic acid since isolated Mn_{12} molecules are very sensitive to the laser radiation.
